# Hemodynamic Changes in the Brachial Artery Induced by Acupuncture Stimulation on the Lower Limbs: A Single-Blind Randomized Controlled Trial

**DOI:** 10.1155/2012/958145

**Published:** 2012-11-25

**Authors:** Masashi Watanabe, Shin Takayama, Atsushi Hirano, Takashi Seki, Nobuo Yaegashi

**Affiliations:** Department of Traditional Asian Medicine, Graduate School of Medicine, Tohoku University, Sendai 980-8575, Japan

## Abstract

Acupuncture is commonly performed at acupoints. No comparisons of quantitative physiological alterations in the brachial artery (BA) induced by the stimulation of different acupoints in the lower limbs have been performed in humans. Therefore, we investigated changes in blood flow volume (BFV) in the BA as an indicator of the physiological effects induced by stimulation at 3 points. Seventy-five healthy participants aged 33 ± 9 years (mean ± SD) were enrolled and randomly assigned to 3 groups; they received stimulation at 3 different points located on the lower limbs: ST36, LR3, and a non-acupoint. Stimulation was performed bilaterally with manual rotation of the needles. Using ultrasonography, BFV was measured continuously from rest to 180 seconds after stimulation. LR3 stimulation significantly increased BFV compared to that before needle insertion. Meanwhile, stimulation at ST36 and the non-acupoint significantly decreased BFV compared to that before needle insertion. Stimulation at LR3 elicited a significant increase in BFV compared to that at ST36 and the non-acupoint. The results suggest that the stimulation of different points on the lower limbs causes distinct physiological effects on BFV in the BA.

## 1. Introduction

 Acupuncture is an important facet of traditional Chinese medicine [1]. Acupuncture points, called acupoints, are gateways located on meridians that can restore the flow of *qi* [2]. The treatment of specific acupuncture points depends on the particular diagnosis [3]. The acupuncture theory is based on the meridian system, which connects acupoints to the organs. However, it has been difficult to verify the meridian and acupoint structures as well as and the connections between organs and meridians. The Zusanli (ST36) acupoint is located on the stomach meridian. This acupoint is known to be effective for the treatment of digestive system disorders; it improves digestive function and decreases abdominal pain [1, 4]. The Taichong (LR3) acupoint is located on the liver meridian; stimulation at this point is known to regulate liver function [1, 4]. One of the functions of the liver is to regulate the free movement of qi. Stagnation of liver qi may impede blood circulation [4] and cause frequent limb chills. However, there is currently no evidence supporting the findings mentioned above.

Blood flow volume (BFV) is an important index for representing the condition of organs and tissues. Therefore, we employed BFV as a quantitative indicator of the effects of traditional interventions on the human body. There are some reports in the literature about the hemodynamic responses to acupuncture stimulation at several acupoints [5–9]. Acupuncture is reported to be effective for treating Raynaud syndrome [10] and chills in the hand [11] as well as improving blood circulation in the forearm. However, these reports only show changes in temperature or blood flow at the skin surface. We previously reported the effects of acupuncture at a single acupoint on the BFV of the upper limb in humans, measured by ultrasonography [12, 13]. In addition, we reported the effects of acupuncture on BFV in the superior mesenteric artery by acupuncture at different acupoints in the lower limbs [14]. To our knowledge, no study has reported changes in the BFV of the brachial artery (BA) induced by acupuncture stimulation at different acupoints on the lower limbs. Therefore, this study aimed to clarify the effects of acupuncture stimulation at 2 different acupoints, ST36 and LR3, and a non-acupoint on the hemodynamics in the BA of healthy participants.

## 2. Methods

### 2.1. Participants

 Seventy-five healthy adult volunteers (45 men and 30 women) aged 33 ± 9 years (mean ± SD; range 20–53 years) were enrolled in this study. No participants had cardiovascular disease. None of the participants took any medicine 1 month before the experiment. All participants were examined after fasting and abstaining from alcohol and caffeine for at least 3 hours. The study protocol was approved by the Ethics Committee of Tohoku University, Graduate School of Medicine. All participants provided written informed consent prior to the beginning of the experiment.

### 2.2. Setting

**Figure 1:**
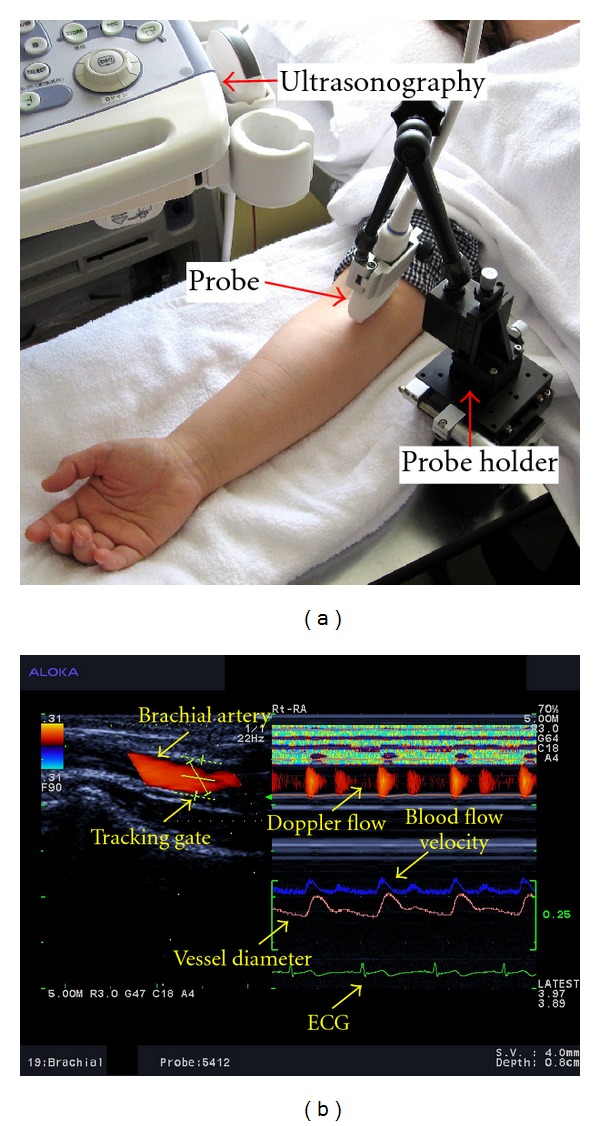
(a) An ultrasonographic measurement of the brachial artery with a special probe holder (MP-PH0001®, Hitachi-Aloka Medical Ltd., Tokyo, Japan). (b) Hemodynamic data obtained by ultrasonography. The left image shows the vessel image and position of the tracking gate of the brachial artery. The right image shows the changes in vessel diameter, Doppler flow, and blood flow velocity determined by an automated edge detection device and computer analysis software (e-tracking system®, Hitachi-Aloka Medical Ltd., Tokyo, Japan).

 The participants rested in the supine position in a quiet, air-conditioned room (temperature 25-26°C). Three monitoring electrocardiographic electrodes were attached to the anterior part of the chest of each participant. Four electrodes for impedance cardiography (ICG) (Bioz ICG Module, Dash 3000®, GE Healthcare, USA) were placed at the base of the neck and the level of the xiphoid process in the midaxillary line. ICG utilizes 4 dual sensors on the neck and chest to apply low-amplitude high-frequency alternating electrical current to the participant's thorax. Pulsatile changes in BFV and velocity are measured as changes in impedance. These changes are subsequently synchronized with the electrocardiogram to automatically calculate hemodynamic parameters such as stroke volume and the cardiac index (CI) [15]. ICG is a noninvasive monitoring method that allows the measurement of the CI according to the changes in thoracic resistance that result from variations in intrathoracic BFV [16, 17]. The CI was calculated on the basis of the stroke volume, heart rate, and body surface area measured by ICG [18]. The systemic vascular resistance index (SVRI) was calculated using the CI and blood pressure. Blood pressure was measured with an oscillometer (BP-608 Evolution II®, Colin Healthcare Co. Ltd., Kyoto, Japan) on the left upper arm. BA hemodynamics was measured by an ultrasonograph (Prosound *α*10®, Hitachi-Aloka Medical Ltd., Tokyo, Japan) on the right arm; this system contains a high-resolution linear array transducer (13 MHz) and a computer-assisted analysis software (e-tracking system® Hitachi-Aloka Medical Ltd., Tokyo, Japan). The software automatically detected the vessel edge and measured the vessel diameter and BFV continuously [19]. The combination of ultrasonography and pulsed Doppler enables the noninvasive investigation of the blood flow in small vessels such as the coronary, splenic, and adrenal arteries as well as the superior mesenteric artery and BA [20]. The right arm was fixed, and the right BA was scanned longitudinally where the vessel diameter and Doppler wave readings were stable. The transducer was fixed in a special probe holder (MP-PH0001®, Hitachi-Aloka Medical Ltd., Tokyo, Japan) at the site where the clearest B-mode image of the anterior and posterior vessel wall was obtained (Figure 1). Care was taken to avoid compressing the artery. The BA diameter was monitored automatically when the tracking gate was placed on the intima of the vessel. The waveform of the changes of vessel diameter over the cardiac cycle was displayed in real time using the e-tracking system® (Figure 1). To obtain accurate measurements, the Doppler angle was maintained at 60° or less [21, 22]. BFV was calculated automatically as the Doppler flow velocity (corrected for the angle) multiplied by heart rate and vessel cross-sectional area [21–23]. To ensure consistent images were obtained, the probe was maintained in the same position throughout the test using a special holder. The e-tracking system® automatically measured changes of vessel diameter with a precision of 0.01 mm. The use of this system avoids operator bias, increases reproducibility, and improves accuracy. The system and software were developed to measure flow-mediated dilatation (FMD), which is usually measured at the BA [24, 25]. Ultrasonography is a noninvasive method for evaluating blood flow velocity. Blood flow changes rapidly in the arteries of the extremities, especially in the peripheral arteries [26]. Changes in venous return due to respiration cause oscillations in stroke volume and blood pressure [27]. Thus, the arterial pulse should be modified by breathing [28]. Therefore, the participants were asked to breathe every 6 seconds during the test, and hemodynamic parameters were calculated as the average values of each 6-second period to minimize the influence of respiration. The following hemodynamic parameters were determined: (1) blood pressure (mmHg), (2) heart rate (beats/min), (3) cardiac index (L·min^−1^·m^−2^), and (4) blood flow volume (mL·min^−1^·m^−2^).

### 2.3. Study Protocol

**Figure 2:**
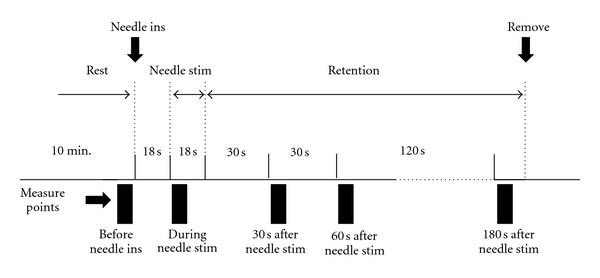
Experimental diagram. Before needle insertion (ins) and stimulation (stim) bilaterally at ST36, LR3, and the non-acupoint. After the needles were inserted, needle stimulation was applied for 18 s with manual rotation. The needles were retained for 180 s after needle stimulation and then removed. Hemodynamic parameters were measured before needle insertion, during, and 30, 60, and 180 s after needle stimulation. min.: minutes, s: seconds.

**Figure 3:**
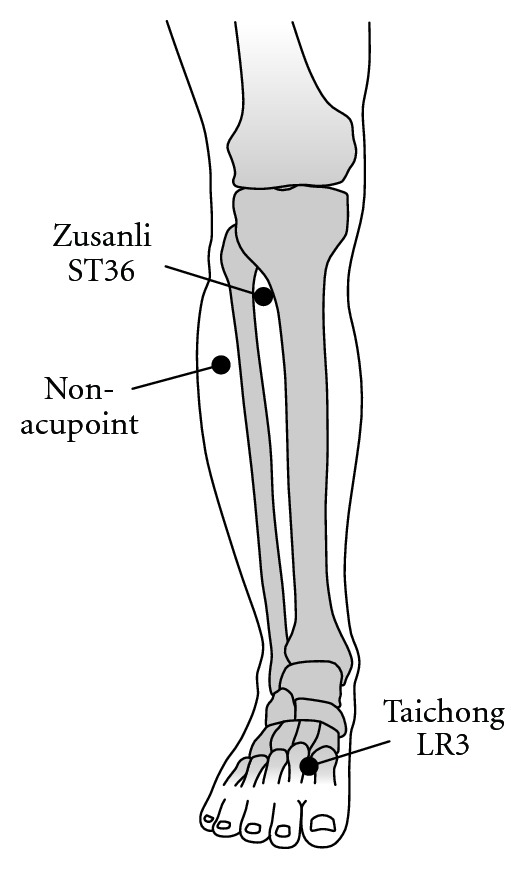
Needle positions of ST36, LR3, and the non-acupoint.

 A single-blind randomized controlled trial was performed. The experimental outline of the study is shown in Figure 2. All participants (*n* = 75) were randomly assigned to 1 of 3 groups receiving the following treatments: (1) needle stimulation at ST36 (*n* = 25, Zusanli: located on the lower leg, 3 units below the lateral “eye” of the knee, approximately 1 finger width lateral to the tibia [29]) (Figure 3); (2) needle stimulation at LR3 (*n* = 25, Taichong: located on the foot, 1.5–2 units above the web between the first and second toes [29]) (Figure 3); (3) needle stimulation at a non-acupoint (*n* = 25: located on the lower leg, 3 units lateral to and below ST36, midpoint of the stomach and gallbladder meridian) (Figure 3). The participants had no knowledge about acupuncture or acupoints.

After the ultrasonograph was positioned, the participants rested in the supine position for 10 minutes. We then measured the BA hemodynamics, blood pressure, heart rate, and the CI at rest (i.e., before needle insertion), during needle stimulation, and 30, 60, and 180 seconds after needle stimulation. Needle stimulation was performed by a licensed acupuncturist. Disposable fine stainless-steel needles (0.16 mm diameter, 40 mm length, Seirin Co. Ltd., Shizuoka, Japan) were inserted bilaterally on the target point and maintained at a depth of 10 mm during the test to ensure they reached the muscle. After the needles were inserted, the stimulation was performed for 18 seconds by rotating the needles <90° manually. The needles were retained for the duration of the test following stimulation and were then removed.

### 2.4. Statistical Analysis

 Statistical analysis was performed with PASW software (version 17.0, SPSS Japan Inc., Tokyo, Japan). Comparisons between the ST36, LR3, and non-acupoint groups were performed by two-way analysis of variance (ANOVA) with a post hoc Tukey test. Repeated-measures one-way ANOVA with a post hoc Dunnett's test were used for statistical comparison between pre- and post-needle insertion values. Mean values between groups were compared using the Kruskal-Wallis H test. The level of statistical significance was set at *P* < 0.05. Individual variations were analyzed as percent changes.

## 3. Results

### 3.1. Participants

 One participant assigned to the LR3 group was excluded from the analysis because of arrhythmia. There were no significant differences among the clinical profiles of the 3 groups (Table 1).

### 3.2. Data Summary

Table 2 summarizes the hemodynamic measurements performed before and after needle insertion in the ST36, LR3, and non-acupoint groups. Stimulation at ST36 elicited a decrease in BFV in the BA during needle stimulation compared with that before needle insertion. The basal values of BFV in the BA were not significantly different among the 3 groups. Similarly, no significant differences were observed in any parameters measured at rest before needle insertion.

### 3.3. Blood Pressure

No significant differences were observed among the 3 groups or with respect to the percent change in systolic or diastolic blood pressure in each test before and after needle insertion. 

### 3.4. Heart Rate

No significant differences were observed among the 3 groups with respect to the percent change in heart rate in each test. Regarding the percent change in heart rate, stimulation at LR3 elicited a significant decrease in heart rate measured during needle stimulation compared to that before needle insertion. The heart rate tended to decrease during needle stimulation.

### 3.5. Cardiac Index

 No significant differences were observed among the 3 groups with respect to the percent change in the CI in each test. Regarding the percent change in the CI, the stimulation at the non-acupoint elicited a significant increase in the CI in the BA 180 seconds after needle stimulation compared to that before needle insertion.

### 3.6. Systemic Vascular Resistance Index

**Figure 4:**
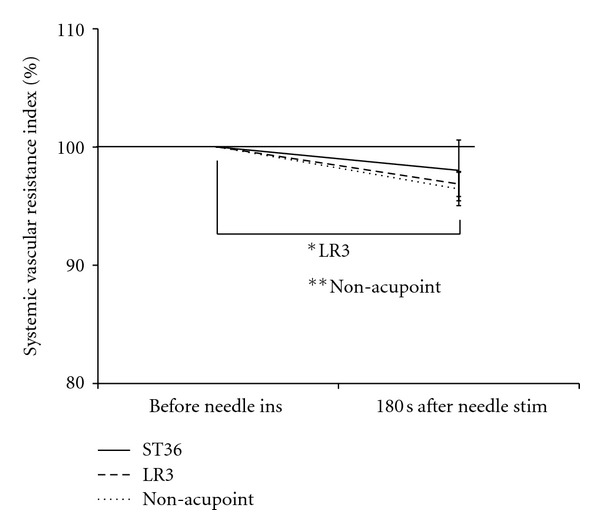
Percent change in the systemic vascular resistance index before needle insertion (ins) and at stimulation (stim) at ST36, LR3, and the non-acupoint. Values represent means and standard errors (SEM). ^∗, ∗∗^Significantly different (*P* < 0.05, *P* < 0.01, resp.) relative to the resting condition in each test setup.

No significant differences were observed among the 3 groups with respect to the percent change in SVRI in each test (Figure 4). Regarding the percent change in SVRI, the stimulation at LR3 and the non-acupoint elicited a significant decrease in SVRI in the BA 180 seconds after needle stimulation compared to that before needle insertion.

### 3.7. Blood Flow Volume of the Brachial Artery

**Figure 5:**
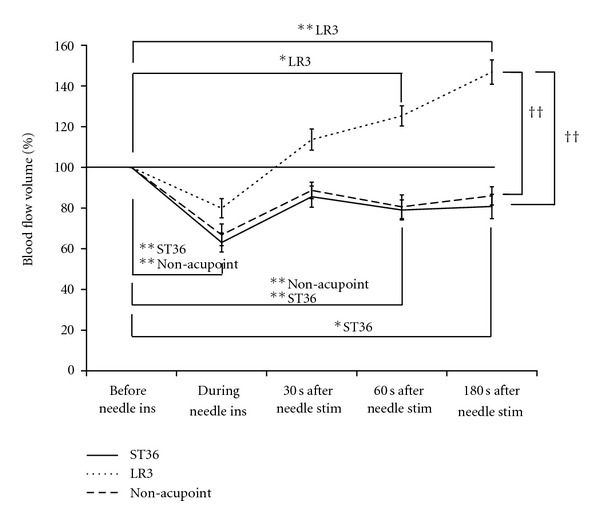
Percent change in blood flow volume in the brachial artery before needle insertion (ins) and during and after needle stimulation (stim) at ST36, LR3, and the non-acupoint. The values represent means and corresponding standard errors (SEM). ^∗, ∗∗^Significant difference (*P* < 0.05 and *P* < 0.01, resp.) relative to the blood flow volume before needle insertion at LR3, ST36, or the non-acupoint. ^††^Significant difference (*P* < 0.01) between the LR3 and ST36/non-acupoint groups.

Figure 5 shows the percent change in BFV in the BA in each test. The percent change in BFV in the BA was significantly different between the LR3 group and the other 2 groups. The stimulation at LR3 elicited a significant increase in BFV in the BA 60 and 180 seconds after needle stimulation compared to that before needle insertion. Meanwhile, the stimulation at ST36 elicited a significant decrease in BFV in the BA during and 60 and 180 seconds after needle stimulation compared to that before needle insertion. Furthermore, stimulation at the non-acupoint elicited a significant decrease in BFV in the BA during and 60 seconds after needle stimulation compared to that before needle insertion.

## 4. Discussion

To our knowledge, this is the first study comparing the differences in BFV in the BA induced by needle stimulation at 3 acupoints as assessed by ultrasonography. The results show that BFV in the BA increased significantly after needle stimulation at LR3, whereas it decreased significantly after needle stimulation at ST36 and the non-acupoint. Furthermore, after needle stimulation at the non-acupoint, the CI increased significantly while SVRI decreased significantly. These results indicate that the physiological effects on the BFV in the BA triggered by needle stimulation vary depending on the acupoint stimulated. Needle stimulation at the lower limbs is known to elicit systemic visceral responses via supraspinal reflexes [30–32]. However, visceral responses vary, as observed in the present study.

Stimulation at ST36 and the non-acupoint elicited significant decreases in BFV in the BA during needle stimulation compared to that before needle insertion. The physiological mechanism involved in the decrease in BFV in the BA induced by the stimulations applied to the skin is a peripheral vascular resistance caused by an instantaneous increase in sympathetic tone [33]. During needle stimulation, heart rate tended to decrease without a change in the CI. Previous studies report that the mechanism of the acupuncture induced decrease in heart rate is based on somatoautonomic reflexes, which occur via the cardiac sympathetic nerves [34–36]. These mechanisms are involved in the supraspinal reflex.

Stimulation at LR3 elicited a significant increase in BFV in the BA 60 and 180 seconds after needle stimulation compared to that before needle insertion. In addition, for LR3 stimulation, a systemic reaction was elicited such that the SVRI decreased significantly 180 seconds after needle stimulation without any changes in blood pressure, heart rate, or the CI compared to those before needle insertion. This reaction suggests that the increase of BFV in the BA after stimulation at LR3 depends on the decrease of SVRI. A previous report suggests that the changes in BFV in the BA caused by needle stimulation at LR3 depend on the decrease of the resistive index of the peripheral artery after needle stimulation [13]. This report supports the relationship between the increase of BFV in the BA and the decrease of SVRI observed in the present study. A previous study found that adrenal sympathetic nerve activity and catecholamine secretion rate increase as a result of stimulating the hind limb of rats [30, 37–39]. In addition, vasodilation is reported to be elicited via adrenalin beta-2 receptor activation [40]. However, in the present study, the BFV of the BA decreased after needle stimulation at the non-acupoint with the decrease of SVRI. This result may suggest that BFV increased in other organs but not in the BA and that the shift of BFV to other organs is induced by needle stimulation. Similar speculation can be applied to the reaction with ST36 stimulation. However, further investigations are required to clarify these reactions.

The physiological reactions mentioned earlier may support the idea that stimulations at ST36, LR3, and the non-acupoint have different impacts on the autonomic nervous system, which regulates BFV in the BA. A recent report also suggested that opposite cardiovascular effects were shown in the response to stimulation of LR3 and ST36 in rats [41]. We can deduce that the various reactions mediated by the autonomic nervous system depend on the characteristics of a specific stimulation, such as the site, intensity, and duration. The 3 points used in this study (ST36, LR3, and the non-acupoint) are located along the boundary of the same dermatomes, and their innervations are complex [42]. Therefore, each point likely has different sensitivities to stimulation. Among other factors, differences in signal transmission via afferent and efferent fibers depend on anatomical structures such as the dermatomes and myotomes as well as the innervation and thickness of the muscle and skin at the stimulation points.

 The present study was a human observational study. Measuring sympathetic and parasympathetic activities in human subjects is invasive and difficult. Therefore, we observed the effects of stimulation at 2 acupoints and 1 non-acupoint on BFV and speculated on the mechanism using previous experimental studies as a basis. In a previous study with 25 participants per group, we reported the changes in BFV in the BA measured by ultrasonography when acupuncture was performed at a single acupoint [12, 13]. According to these results, we enrolled 25 participants in each of the 3 groups in the present study. The use of ultrasound methods to assess FMD has a good reproducibility [43–45]. However, a previous study evaluating the reproducibility of FMD indicates that 62 subjects per group are required to detect a treatment difference of 2 FMD% with a probability of 0.05 and a power of 0.80 in a parallel design comparing intergroup changes [46]. Therefore, the small sample size of each group may be a limitation of the present study. The findings of this study suggest that acupuncture induces endothelium-dependent FMD, providing an interesting pathophysiological basis for the explanation of observed blood flow changes.

## 5. Conclusions

 The present study revealed that BFV in the BA increased after needle stimulation at LR3 without changes in the CI. Needle stimulation at ST36 and the non-acupoint affected the hemodynamics of the BA. These physiological effects may be dependent on acupoint location.

## Figures and Tables

**Table 1:** Clinical profiles by group.

Profile	Stimulation points		
	ST36	LR3	Non-acupoint
Person	25	24	25
Sex (men : women)	15 : 10	14 : 10	15 : 10
Age (year)	31.0 ± 9.7	34.6 ± 8.4	33.1 ± 8.3
Height (cm)	166.9 ± 9.2	167.7 ± 8.9	165.5 ± 8.1
Weight (kg)	64.0 ± 12.1	61.7 ± 10.9	60.3 ± 9.5
Body surface area (m^2^)	1.71 ± 0.19	1.70 ± 0.19	1.66 ± 0.15

**Table 2:** Summary of measured hemodynamic parameters.

Parameter/test condition	Before needle ins	During needle stim	30 s after needle stim	60 s after needle stim	180 s after needle stim
Systolic blood pressure (mm Hg)					
ST36	116.1 ± 11.0				116.2 ± 10.2
LR3	114.7 ± 12.7				112.1 ± 12.3
Non-acupoint	117.5 ± 15.5				116.0 ± 14.0
Diastolic blood pressure (mm Hg)					
ST36	69.1 ± 8.0				69.0 ± 8.6
LR3	68.2 ± 9.1				66.1 ± 9.0
Non-acupoint	70.4 ± 12.2				70.0 ± 11.0
Heart rate (beats/min)					
ST36	66.2 ± 10.2	64.1 ± 10.7	67.0 ± 10.7	66.4 ± 10.5	68.0 ± 11.7
LR3	63.4 ± 9.0	60.4 ± 7.8	63.0 ± 9.1	63.4 ± 9.1	63.6 ± 9.1
Non-acupoint	65.0 ± 9.5	62.6 ± 10.5	64.5 ± 9.4	64.8 ± 9.6	64.8 ± 10.4
Cardiac index (L·min^−1^·m^−2^)					
ST36	3.0 ± 0.7	3.0 ± 0.6	3.0 ± 0.6	3.0 ± 0.7	3.0 ± 0.7
LR3	2.7 ± 0.4	2.7 ± 0.4	2.7 ± 0.5	2.7 ± 0.5	2.7 ± 0.5
Non-acupoint	2.7 ± 0.4	2.7 ± 0.4	2.7 ± 0.4	2.8 ± 0.4	2.8 ± 0.4
Systemic vascular resistance index (dyne sec/cm^5^ m^2^)					
ST36	2390 ± 513				2347 ± 556
LR3	2507 ± 429				2414 ± 431
Non-acupoint	2602 ± 654				2522 ± 586
Blood flow volume in brachial artery (mL·min^−1^·m^−2^)					
ST36	55.4 ± 29.3**	33.4 ± 13.1**	48.0 ± 21.5	44.0 ± 20.8	45.5 ± 25.4
LR3	38.9 ± 25.9	29.8 ± 20.6	41.8 ± 25.1	47.7 ± 30.7	54.8 ± 32.6
Non-acupoint	59.0 ± 52.4	37.7 ± 31.0	57.8 ± 50.6	48.0 ± 40.5	53.3 ± 40.7

The values represent the mean ± SD. **Significant difference (*P* < 0.01) in the blood flow volume in the brachial artery between before needle insertion (ins) and during needle stimulation (stim) at ST36.
